# Controllable Fabrication and Optical Properties of Uniform Gadolinium Oxysulfate Hollow Spheres

**DOI:** 10.1038/srep17934

**Published:** 2015-12-16

**Authors:** Fashen Chen, Gen Chen, Tao Liu, Ning Zhang, Xiaohe Liu, Hongmei Luo, Junhui Li, Limiao Chen, Renzhi Ma, Guanzhou Qiu

**Affiliations:** 1 School of Materials Science and Engineering, Central South University, Changsha, Hunan 410083, China; 2 Department of Chemical Engineering, New Mexico State University, Las Cruces, New Mexico 88003, United States; 3 State Key Laboratory of High Performance Complex Manufacturing and School of Mechanical and Electronical Engineering, Central South University, Changsha, Hunan 410083, China

## Abstract

Uniform gadolinium oxysulfate (Gd_2_O_2_SO_4_) hollow spheres were successfully fabricated by calcination of corresponding Gd-organic precursor obtained via a facile hydrothermal process. The Gd_2_O_2_SO_4_ hollow spheres have a mean diameter of approximately 550 nm and shell thickness in the range of 30–70 nm. The sizes and morphologies of as-prepared Gd_2_O_2_SO_4_ hollow spheres could be deliberately controlled by adjusting the experimental parameters. Eu-doped Gd_2_O_2_SO_4_ hollow spheres have also been prepared for the property modification and practical applications. The structure, morphology, and properties of as-prepared products were characterized by XRD, TEM, HRTEM, SEM and fluorescence spectrophotometer. Excited with ultraviolet (UV) pump laser, successful downconversion (DC) could be achieved for Eu-doped Gd_2_O_2_SO_4_ hollow spheres.

Hollow spheres have been attracting great attention due to their superior properties such as high specific surface area, low density, high permeability and therefore show promising potential applications in various fields such as lithium batteries, catalysis and sensing, drug controlled release and delivery, and photonic building blocks, etc^1^,^2^,^3^,^4^,^5^,^6^. Plenty of chemical and physicochemical strategies such as Ostwald ripening^7^, Kirkendall diffusion^8^, chemically induced self-transformation^9^, template-assisted synthesis^10^, and spray drying followed by annealing^11^,^12^ have been applied for the design and controlled fabrication of various micro/nanospheres with hollow interiors. In particular, template-assisted synthesis has been demonstrated to be the most effective and versatile synthesis method. The templates can be generally divided into hard templates^13^,^14^,^15^ and soft templates^16^,^17^,^18^, which have been widely used to fabricate hollow spheres. Among them, biomolecules, as attractive templates for the synthesis of metal and inorganic compound nanostructures, have been exploited for the precise control of the size and shape of various micro/nanomaterials, owing to the well-defined chemical and structural heterogeneity^19^,^20^,^21^,^22^. In spite of these pioneering work, it is still challenging and imperative to exploit an efficient but simple way for the synthesis of hollow spheres.

Rare earth oxysulfate (RE_2_O_2_SO_4_) have aroused great interest in recent years due to the unique magnetic^23^ and luminescent properties^24^,^25^ as well as significant applications in large volume oxygen storage^26^,^27^. RE_2_O_2_SO_4_ is also an important matrix compound for luminescent rare-earth ions to fabricate downconversion (DC) or upconversion (UC) phosphors due to the incompletely filled 4*f* electron shell of rare-earth ions^28^,^29^,^30^. RE_2_O_2_SO_4_ could be synthesized by the thermal decomposition of the corresponding hydrous sulfates (RE_2_(SO_4_)_3_∙nH_2_O), layered rare-earth hydroxides intercalated with dodecyl sulfate (DS) ions, and layered rare-earth hydroxylsulfate (RE_2_(OH)_4_SO_4_∙nH_2_O)^31^,^32^,^33^,^34^. Nevertheless, the size and morphology of RE_2_O_2_SO_4_ products prepared by the above methods are not well controlled and no particular shape or uniform size can be achieved. Recently, we reported a unique synthetic process to prepare Y_2_O_2_SO_4_ hollow structure, which was mainly intended for the use of photoluminescence host materials^35^.

For Gd_2_O_2_SO_4_, due to its unique half-filled outer electron shell in rare-earth elements, it is promising in combining magnetic and luminescent properties. A peculiar hollow structure further endows Gd_2_O_2_SO_4_ to be a multifunctional nanomaterial for biomedical applications, such as magnetic resonance imaging, drug delivery host carriers and diagnostic analysis. This brings far-reaching impact than the availability of Y_2_O_2_SO_4_ hollow structure. Herein, we present a facile biomolecule-assisted route to prepare uniform Gd_2_O_2_SO_4_ hollow spheres via the calcination of corresponding spherical Gd-organic precursor obtained by using L-cysteine (Cys) as a biomolecule template. The size and morphology of as-prepared Gd_2_O_2_SO_4_ hollow spheres can be deliberately controlled by adding different surfactants with varied amount. The formation process of the hollow spheres is elucidated by monitoring the species change and crystal structure evolution with elevated annealing temperature. Eu-doped Gd_2_O_2_SO_4_ hollow spheres have also been successfully synthesized and the luminescence properties of as-prepared products were studied in detail.

## Results

**Figure 1:**
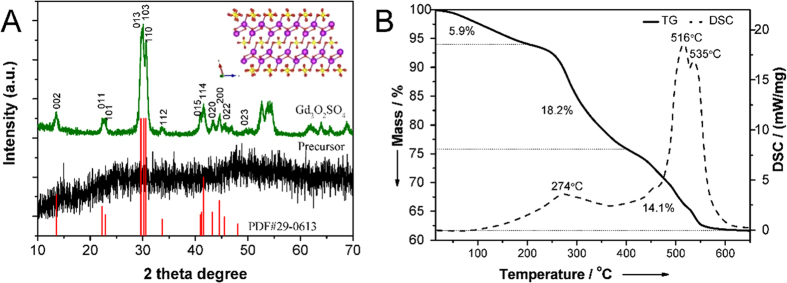
(**A**) XRD patterns of as-prepared Gd-organic precursor and corresponding Gd_2_O_2_SO_4_. The inset depicts the corresponding crystal structure of Gd_2_O_2_SO_4_. The Gd, O, and S species are represented by violet, red, and yellow balls, respectively. (**B**) TG and DSC curves of as-prepared Gd-organic precursor annealing from 25 to 650 °C at a heating rate of 10 °C min^−1^ in air.

X-ray diffraction (XRD) was carried out to illuminate the change and evolution of chemical composition and crystal structures. Figure 1A shows the XRD patterns of the Gd-organic precursor and corresponding Gd_2_O_2_SO_4_ obtained by calcination at 600 °C for 2 h. No diffraction peaks were verified, indicating the initial precursor with broad featureless peaks was amorphous or non-crystalline. After annealing at 600 °C for 2 h, the precursor was converted into a single phase of Gd_2_O_2_SO_4_, and no other impurity phases can be observed. All the reflections can be indexed to the literature values (JCPDS 29–0613). The crystal structure of Gd_2_O_2_SO_4_ can commonly be depicted as an alternative stacking of Gd_2_O_2_^2+^ and anion groups of sulfate (SO_4_^2−^) layers along the a-axis, as shown in the inset of Figure 1A. The Gd_2_O_2_^2+^ layer consists of [GdO_4_] tetrahedra linked together by shared of edges. Every [SO_4_] tetrahedra unit is coordinated with two Gd atoms^36^. The thermal decomposition behaviors of Gd-organic precursor was investigated in the temperature range of 25–650 °C at a heating rate of 10 °C min^−1^ in air. As shown in Figure 1B, the weight loss in the temperature range from 25 to 200 °C was about 5.9% by mass, which can be associated with evaporation of physically absorbed water and organic residues on the Gd-organic precursor surfaces. The subsequent weight loss took place rapidly at a much higher temperature range. The continuous stages of weight loss in the range of 200 to 600 °C were 18.2% and 14.1% by mass. The tremendous decrease of weight can be attributed to the oxidation or combustion of the initial precursor and crystallization into Gd_2_O_2_SO_4_. Corresponding to the two remarkable mass loss, the DSC curve of the sample displayed three major exothermal peaks in the gravimetric gain region centered at 274 °C, 516 °C and 535 °C respectively. As shown in the TG curve, little weight change can be observed at temperatures higher than 600 °C, suggesting that the relatively stable compound was obtained. Therefore, the hydrothermal products were annealed at 600 °C for the crystallization of Gd_2_O_2_SO_4_ hollow spheres.

**Figure 2:**
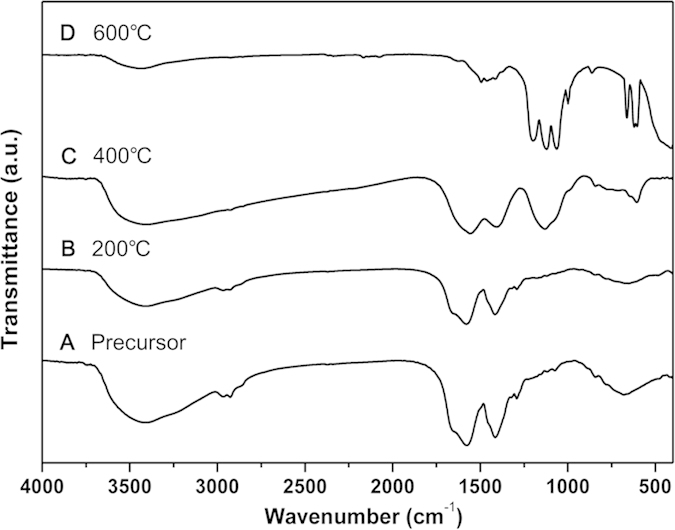
FT-IR spectras of the Gd-organic precursors (**A**) and the powders after calcinating at 200 °C (**B**), 400 °C (**C**) and 600 °C (**D**) for 2 h.

Fourier transform infrared (FT-IR) spectroscopy was employed to investigate the structural and functional group information of the Gd-organic precursors and powders calcined at different temperatures. As shown in the Figure 2A, the FT-IR spectra reveal the existence of absorbed water, crystal water, hydroxyl groups (~3410 cm^−1^ and 1640 cm^−1^), carbonates anions (~1580 cm^−1^ and 1415 cm^−1^) and sulfates anions (~680 cm^−1^) in the Gd-organic precursors^37^. The weak peaks at 2965 cm^−1^ and 2927 cm^−1^ are assigned to the -C-H vibration mode of -CH_2_^38^. As the temperature of calcination increasing to 200 °C and 400 °C, the broaden band at 3410 cm^−1^ becomes weaker and weaker while the small peak at 1640 cm^−1^ disappears at 400 °C, which can be attributed to the removal of absorbed water and crystal water from the Gd-organic precursors. A similar behavior of carbonates absorption bands at 1580 cm^−1^ and 1415 cm^−1^ can be observed, suggesting that the carbonate anions in the precursors decomposed or vaporized with increasing the temperature. These results are in good agreement with the results of TG-DSC analysis. Both the broaden band at 3410 cm^−1^ and carbonates absorption bands are significantly reduced at a higher calcination temperature of 600 °C; while a broaden sulfates absorption band at 1130 cm^−1^ appears at 400 °C and splits into three narrow and sharp peaks at 1198 cm^−1^, 1121 cm^−1^ and 1063 cm^−1^ at 600 °C. The broaden sulfates absorption band at 680 cm^−1^ in the precursors becomes weaker and splits into three narrow and sharp peaks at 663 cm^−1^, 621 cm^−1^ and 603 cm^−1^ in the final products. These two group of narrow and sharp sulfates absorption bands are assigned to the deformation vibrations and the asymmetric stretching of SO_4_^2−^ anions, respectively^39^. These results are in accordance with those obtained from TG-DSC, XRD patterns in Figure S1 and ICP analysis in Table S1, illustrating the composition and structural evolution of the Gd_2_O_2_SO_4_ products.

**Figure 3:**
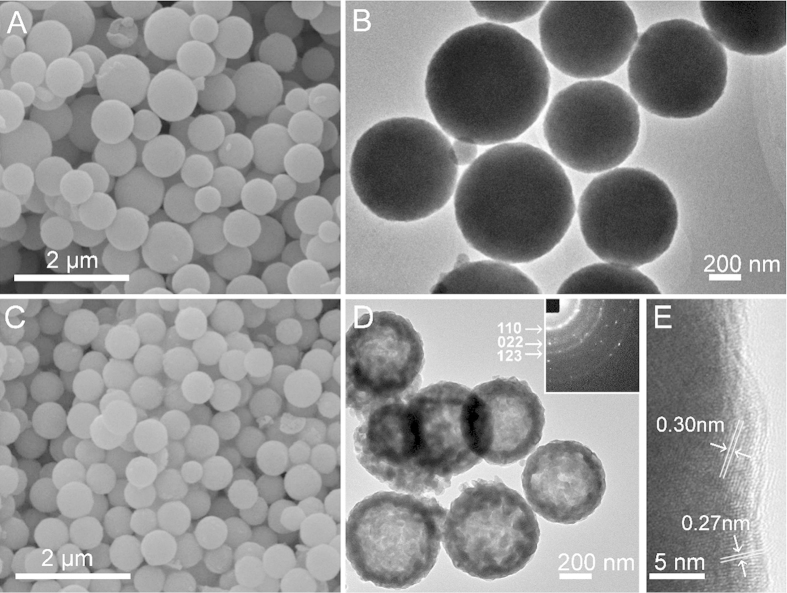
(**A**) SEM and (**B**) TEM images of spherical Gd-organic precursors. (**C**) SEM and (**D**) TEM images of Gd_2_O_2_SO_4_ hollow spheres. The inset in (**D**) is corresponding SAED pattern; (**E**) HRTEM image of Gd_2_O_2_SO_4_ hollow sphere.

Scanning electron microscopy (SEM) and transmission electron microscopy (TEM) were employed to characterize the sizes and morphologies of as-prepared products. Figure 3A,B show the spherical Gd-organic precursors with a smooth surface and an average size of approximately 650 nm. After calcinating the Gd-organic precursors at 600 °C for 2 h, as shown in Figure 3C,D, Gd_2_O_2_SO_4_ hollow spheres with relatively rough surfaces were obtained. The average diameter of the hollow spheres was estimated to be approximately 550 nm, as shown in Figure S2, with a slightly decreasing in comparison with that of the precursor, implying the tendency to shrink after calcination. The strongly contrast between the dark periphery and greyish center of Gd_2_O_2_SO_4_ spheres reveals that these spheres were of hollow structures, and the shell thickness was about 60 nm. The inset in Figure 3D represents a typical selected area electron diffraction (SAED) pattern, which can be indexed to the monoclinic structure of Gd_2_O_2_SO_4_, consistent with the XRD result presented above. Figure 3E displays the corresponding high-resolution TEM (HRTEM) image, in which the lattice fringes were measured to be about 0.27 and 0.30 nm, corresponding to the interplanar spacings between (112) and (013) crystallographic planes, respectively. The current synthetic route could be adopted as a general strategy for the preparation of a series of rare-earth oxysulfate hollow spheres.

L-Cys, as a biomolecule template, possesses abundant functional groups, such as -SH, -NH_2_, and -COOH, which can coordinate to Gd^3+^ and form homogeneous Gd-organic coordination compound on the basis of metal-ligand interaction in the solution^3^,^4^,^35^, leading to the formation of spherical precursors through aggregation and coagulation. Calcination temperature-depended formation mechanism of hollow spheres was investigated in detail as shown in Figure S3. After calcinating the solid spherical Gd-organic precursors at 200 °C for 2 h shown in Figure S3A, dark periphery and slightly greyish center of the spheres could be observed in the product. As the temperature of calcination increasing to 400 °C, the area of the greyish center of the spheres increased. Finally, the spheres with apparent hollow structure were obtained at the calcination temperature of 600 °C. We consider that the formation mechanism of the hollow spheres may involve two steps: First, a dense rigid shell formed in the surface of the solid spheres as the existence of the a large temperature gradient (∆T) along the radial direction at initial stage of calcination^40^. Then in the subsequent calcination, as the adhesion force (*Fa*) surpasses the contraction force (*Fc*), the inner part shrinks outward, a hollow cavity in the center of the spheres were obtained^41^. The organic substances were all burnt out at 600 °C and the Gd-organic precursors were gradually crystallized into Gd_2_O_2_SO_4_ at the peripheries, meanwhile, the hollow structure was formed.

**Figure 4:**
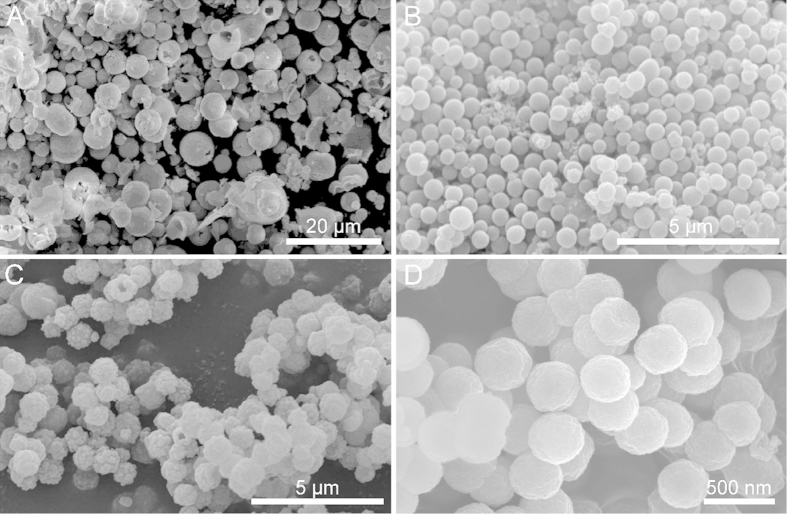
SEM images of as-prepared Gd_2_O_2_SO_4_ hollow spheres obtained by using different surfactants: (**A**) without any surfactants; (**B**) 0.15 g PVP; (**C**) 0.6 g PVP; (**D**) 1 mmol CTAB.

It was generally believed that surfactants played an important role in the control of morphologies and sizes of nanomaterials. Xia *et al*. studied the metal crystal growth kinetic process by using the different surfactants, such as cetyltrimethyl ammonium bromide (CTAB), polyvinylpyrrolidone (PVP), polyethylene glycol (PEG) and so on, to maneuver the surface energies and growth rates for different facets^42^,^43^. The ratio between growth rates of different facets determined the growth habit of a nanocrystal, leading to the formation of different sizes and morphologies of nanomaterial. PVP had been widely introduced into the shape controlled synthesis of nanomaterials, such as and nanowires, nanosheets, nanospheres and so forth^44^,^45^. In this paper, we have studied the effect of surfactants on the synthesis of Gd_2_O_2_SO_4_ hollow spheres. Figure 4A shows the SEM image of as-prepared Gd_2_O_2_SO_4_ without using any surfactants. Although Gd_2_O_2_SO_4_ hollow spheres with broken shell could be observed in the absence of surfactant, the products had a tendency to agglomerate into block, and the size also reached the micrometer range. As shown in Figure 4B, when 0.15 g PVP was introduced into the synthesis of Gd_2_O_2_SO_4_ hollow spheres. The resulting product was mainly uniform spherical particles with smooth surfaces. However, with increasing the amount of PVP to 0.6 g (Figure 4C), the surface of hollow spheres became relatively rough. Thus, 0.3 g PVP was chosen as an optimal amount in the typical synthetic procedure of Gd_2_O_2_SO_4_ hollow spheres. The exact mechanism of the function of PVP on the morphology and size of Gd_2_O_2_SO_4_ hollow spheres is yet to be fully understood, it is believed that the strong interaction between the surfaces of Gd-organic precursors and PVP through coordination bonding with the O and N atoms of the pyrrolidone ring played a major role in determining the product morphology and size^45^. We also found that the CTAB as surfactant has similar functions in the synthesis of Gd_2_O_2_SO_4_ hollow spheres, as shown in Figure 4D. CTAB was used instead of PVP while other synthetic parameters were kept unchanged. The resulting product was mainly uniform Gd_2_O_2_SO_4_ spheres with rough surface and the average size decreased to approximately 350 nm. These results further proved the indispensable role of surfactants in the formation of Gd_2_O_2_SO_4_ hollow spheres.

**Figure 5:**
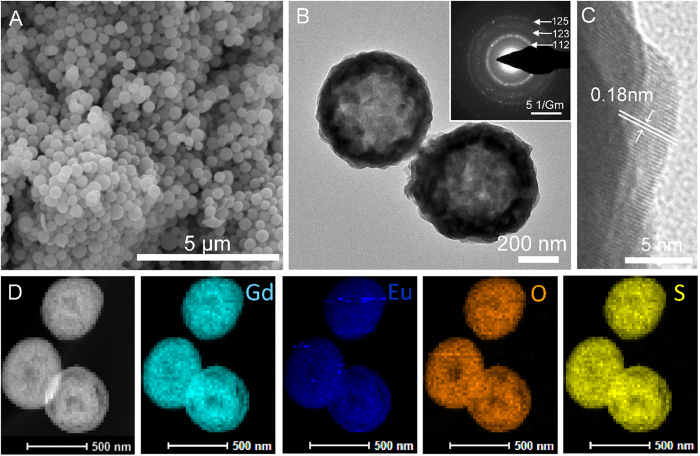
(**A**) SEM and (**B**) TEM images of as-prepared 5% Eu-doped Gd_2_O_2_SO_4_ hollow spheres. Inset is the corresponding SAED pattern. (**C**) HRTEM image of 5% Eu-doped Gd_2_O_2_SO_4_ hollow spheres; (**D**) STEM HAADF and elemental maps of Gd, Eu, O and S of 5% Eu-doped Gd_2_O_2_SO_4_ hollow spheres.

The introduction of other rare-earth ions such as Eu^3+^ ions into Gd_2_O_2_SO_4_ host lattice caused little change both on morphology and crystal phase. As shown in Figure 5, when 5% Eu^3+^ was added into the Gd_2_O_2_SO_4_ host lattice, the morphology of final products, as well as the organic precursor shown in Figure S4, remained unchanged compared with the pure Gd_2_O_2_SO_4_. The crystalline nature of Gd_2_O_2_SO_4_:Eu hollow spheres was confirmed by HRTEM. Figure 5C clearly shows the lattice fringes were measured to be about 0.18 nm, corresponding to the interplanar spacing of (024) crystallographic plane, which fairly well agree with the standard interplanar spacing. The result of X-ray diffraction analyses further proved that the introduction of 5% Eu^3+^ ions into the Gd_2_O_2_SO_4_ host lattice has no significant change on the crystal structure, as show in Figure S5, owing to the same trivalent state and similar ionic radius of Gd^3+^ ions (r_(Gd3+)_ = 0.0938nm) and Eu^3+^ ions (r_(Eu3+)_ = 0.095 nm). The elemental maps of the 5% Eu-doped Gd_2_O_2_SO_4_ hollow spheres obtained on TEM were displayed in Figure 5D, which clearly demonstrates a homogeneous distribution of Gd, Eu, S and O elements. The energy dispersive spectrometer (EDS) spectrum in Figure S6 reveals that the as-obtained product mainly contains Gd, Eu, S and O elements (Au signals were come from the spray-gold treatment to enhance the electrical conductivity of the material). The molar ratio of Eu:Gd was about 3.23:96.77, which was consistent with the ratio of used reagents in synthetic process. The above results confirm that successful doping could be achieved through current synthetic strategy.

## Discussion

**Figure 6:**
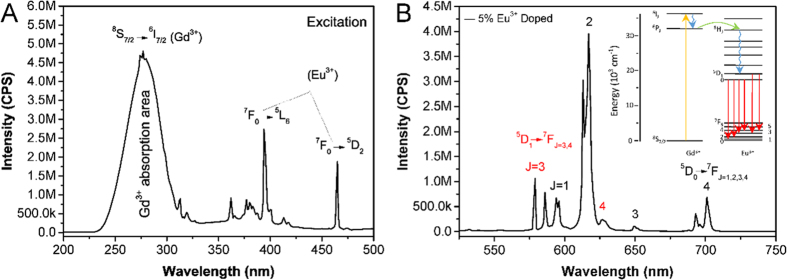
(**A**) Excitation spectrum of 5% Eu-doped Gd_2_O_2_SO_4_ hollow spheres. (**B**) Emission spectrum of 5% Eu-doped Gd_2_O_2_SO_4_ hollow spheres. Inset is corresponding scheme of the energy level and energy transition of 5% Eu-doped Gd_2_O_2_SO_4_ hollow spheres.

The excitation spectra of the 5% Eu-doped Gd_2_O_2_SO_4_ phosphors was recorded in the wavelength range of 200–500 nm at room temperature, as shown in Figure 6A, one can see that a broad absorption band with a maximum at around 270 nm exists, which is resulted from the typical ^8^S_7/2_ → ^6^I_7/2_ transition of the Gd^3+^ ions^46^. Furthermore, other two comparatively weak peaks centered at 394 nm and 465 nm can be respectively assigned to the typical f-f transition of Eu^3+^ ions, corresponding to the ^7^F_0_ → ^5^L_6_ and ^7^F_0_ → ^5^D_2_ transitions^37^. Excitation spectra of the 5% Eu-doped Gd_2_O_2_SO_4_ phosphors was taken by monitoring the wavelength of 617 nm.

The emission spectrums of 5% Eu-doped Gd_2_O_2_SO_4_ under 270 nm light excitation (Figure 6B) demonstrate the characteristic ^5^D_0_ → ^7^F_J_ (J = 1, 2, 3, 4) and ^5^D_1_ → ^7^F_J_ (J = 3, 4) transitions of Eu^3+^ ions, indicating the effective cooperative luminescence between Gd^3+^ and Eu^3+^. The strongest emission which splits into two peaks centered at 613 nm and 617 nm can be attributed to the forced electric dipole ^5^D_0_ → ^7^F_2_ transition of Eu^3+^ ions. All the other emission peaks are easily assigned to the ^5^D_1_ → ^7^F_3_ (579, 586 nm), ^5^D_0_ → ^7^F_1_ (594, 596 nm), ^5^D_1_ → ^7^F_4_ (627 nm), ^5^D_0_ → ^7^F_3_ (649 nm), ^5^D_0_ → ^7^F_4_ (693, 701 nm) transition of Eu^3+^ ions, respectively^47^,^48^,^49^,^50^. In this process, trivalent Gd^3+^ ions, as sensitizer, absorb ultraviolet excitation light and subsequently transfer energy to the neighboring Eu^3+^ ions act as activator, resulting in the overall red emission of Eu^3+^. The detailed energy level and transfer scheme was shown in inset of Figure 6B. Upon excitation by 270 nm, Gd^3+^ ions will be excited into ^6^I_7/2_ state from ground state in the first step and then fast relax from this high excitation state to the ^6^P_J_ state. Secondly, the Gd^3+^ ions in the ^6^P_J_ state can easily transfer the excitation energy to the Eu^3+^ ions (^5^H_J_) because of the energy level match between ^6^P_J_ state and ^5^H_J_ state^50^. Fast non-radiative relaxation from ^5^H_J_ state to the ^5^D_1_ or ^5^D_0_ state occurs. The electron on high excitation ^5^D_1_ and ^5^D_0_ states further relaxes radiatively to the ground-state to generate different wavelength visible emissions. Furthermore, as shown in Figure S7, the emission intensity of the Gd-organic precursors with poor crystallinity can be negligible comparing to the final products with high crystallinity.

## Conclusions

In summary, uniform gadolinium oxysulfate hollow spheres have been successfully achieved by a facile hydrothermal process combining with a calcination of Gd-organic precursors. Based on the experimental results, we found both the amount and the type of surfactants play an important role for the formation of Gd_2_O_2_SO_4_ hollow spheres. Eu-doped Gd_2_O_2_SO_4_ hollow spheres have also been successfully synthesized with little change both on size and crystal phase. Optical properties reveal that the Eu-doped Gd_2_O_2_SO_4_ hollow spheres can be used to down-convert UV light to visible light under the UV excitation. It is expected that the uniform Gd_2_O_2_SO_4_ hollow spheres have potential applications in various research field, such as large volume oxygen storage, drug delivery host carriers, optical/display devices and luminescence probes.

## Methods

All the reagents are of analytical grade and used as starting materials without further purification.

### Preparation of gadolinium oxysulfate hollow spheres

In a typical synthetic procedure of Gd_2_O_2_SO_4_ hollow spheres, 1 mmol of hydrated gadolinium nitrate (Gd(NO_3_)_3_·6H_2_O), 2.0 mmol of L-Cys (L-cysteine) and 0.3 g of PVP (polyvinylpyrrolidone) were dissolved in 20 ml deionized water under vigorous magnetic stirring. Then the resulting solution was transferred into Teflon-lined stainless steel autoclave of 50 ml capacity and maintained at 140 °C for 24 h. After cooling to room temperature naturally, the resulting precipitates were washed with distilled water and anhydrous alcohol for several times, and dried at 50 °C for 4 h. Finally, the precursors can be transformed into Gd_2_O_2_SO_4_ hollow spheres by calcination the Gd-organic precursor at 600 °C for 2 h. Furthermore, the 5% Eu-doped Gd_2_O_2_SO_4_ hollow spheres were also obtained by similar process.

### Characterization

X-ray diffraction patterns were recorded by a D/max2550 VB+ diffractometer with Cu Kα radiation (λ = 0.15405 nm) in the 2θ range of 10°–70°. The morphology of the as-prepared products was examined by a field emission scanning electron microscopy (FE-SEM, Sirion 200) with an accelerating voltage of 15 kV. The energy dispersive spectrometer (EDS) was taken on the SEM. Transmission electron microscopy (TEM) images, selected area electron diffraction (SAED), high-resolution TEM (HRTEM) and the elemental mapping were recorded on a Tecnai G2 F20 transmission electron microscope with an accelerating voltage of 200 kV. Thermogravimetric and differential scanning calorimetry (TG-DSC) were carried out using a simultaneous thermal analysis (STA, NETZSCH STA 449C) in a temperature range of 25–650 °C at a heating rate of 10 °C min^−1^ under an air flow. Fourier transform infrared (FT-IR) spectroscopy were obtained on a Nicolet Nexus 6700 instrument. Baird PS-6 Inductively Coupled Plasma Atomic Emission Spectrometer (ICP-AES) were used to evaluate the element content. The photoluminescence (PL) excitation and emission spectras were obtained on a fluorescence spectrophotometer (Hitachi F-4500) at room temperature.

## Additional Information

**How to cite this article**: Chen, F. *et al*. Controllable Fabrication and Optical Properties of Uniform Gadolinium Oxysulfate Hollow Spheres. *Sci. Rep*. **5**, 17934; doi: 10.1038/srep17934 (2015).

## Supplementary Material

Supplementary Information
