# Supplementary material for: Controllable Fabrication and Optical Properties of Uniform Gadolinium Oxysulfate Hollow Spheres
Source: Sci Rep. 2015 Dec 16;5:17934. doi: 10.1038/srep17934 (PMC4680860; doi:10.1038/srep17934)
Supplement: Supplementary Information [file srep17934-s1.docx]

***Supporting Information for***

**Controllable Fabrication and Optical Properties of Uniform Gadolinium Oxysulfate Hollow Spheres**

Fashen Chen,^a^ Gen Chen,^a,b^ Tao Liu,^a^ Ning Zhang,^a^ Xiaohe Liu,^a,*^ Hongmei Luo,^b^ Junhui Li,^c,*^ Limiao Chen,^a^ Renzhi Ma,^a,*^ Guanzhou Qiu^a^

^a^ School of Materials Science and Engineering, Central South University, Changsha, Hunan 410083, China. Email: liuxh@csu.edu.cn; marenzhi@csu.edu.cn

^b^ Department of Chemical Engineering, New Mexico State University, Las Cruces, New Mexico 88003, United States.

^c^ State Key Laboratory of High Performance Complex Manufacturing and School of Mechanical and Electronical Engineering, Central South University, Changsha, Hunan 410083, China. Email: lijunhui@csu.edu.cn





**Figure S1**. XRD patterns of the Gd-organic precursors after calcinating at 200 °C and 400 °C for 2 h.

|  | Gd/S |
| --- | --- |
| Precursors | 0.885 |
| 200℃ | 0.898 |
| 400℃ | 2.363 |
| 600℃ | 2.263 |

**Table S1**. The atomic ratio between Gd and S of the as prepared product based on ICP-AES analysis.

As the total element content of Gd does not lose during the thermal decomposition of Gd-organic precursors or the crystallization of Gd_2_O_2_SO_4_, the variation of atomic ratio Gd/S can be regarded as another mean to investigate thermal decomposition behaviors of Gd-organic precursor. As shown in **Table S**1, the atomic ratio Gd/S of the powder calcinating at 200 °C changes little compared to the precursors and increases near to the theoretical value 2 at a higher temperature of 400°C. This can be attributed to weight loss of element S caused by the oxidation or combustion of the Gd-organic precursors, which are consistent with the results of TG-DSC analysis. The atomic ratio Gd/S of the powder calcinating at 400 °C was similar to 600 °C, suggesting that the initial crystallization of Gd_2_O_2_SO_4_ was obtained at 400°C and in accordance with the results of XRD patterns shown in **Figure S**1.


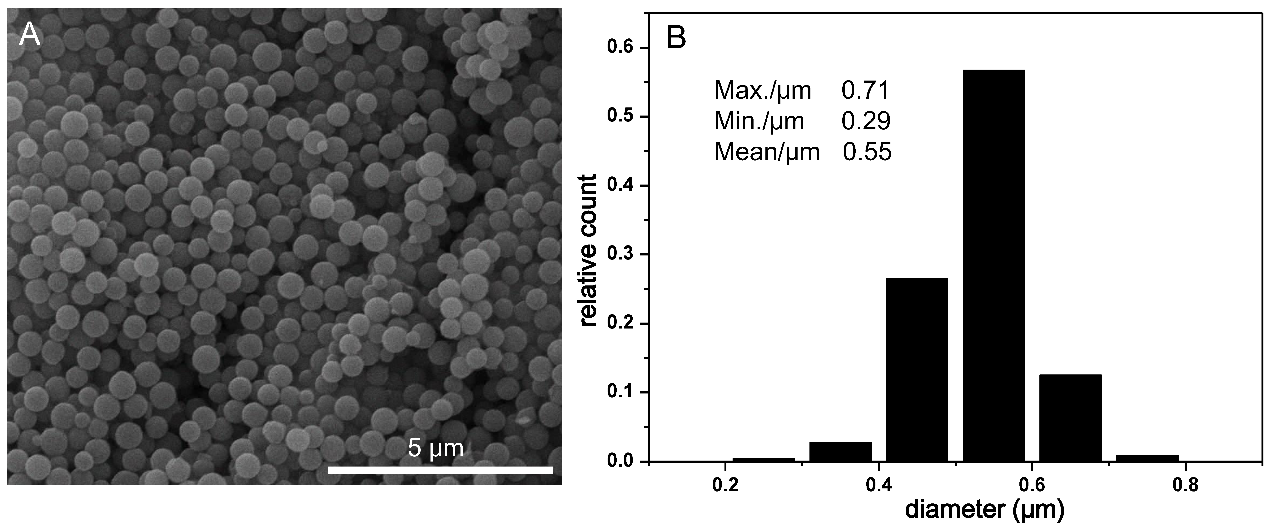


**Figure S2**. Low-magnification SEM image of Gd_2_O_2_SO_4_ hollow spheres (A) and corresponding static particles size distribution (B).


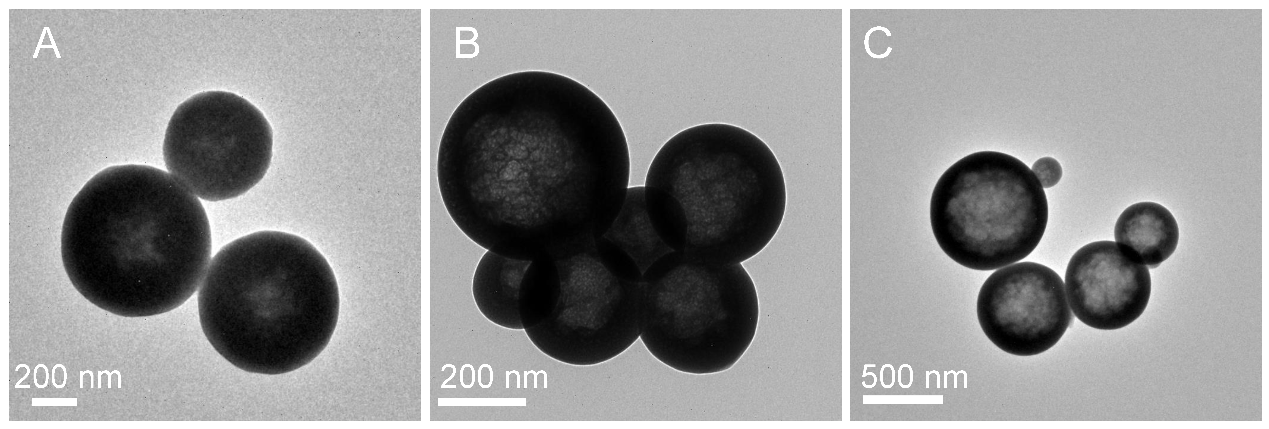


**Figure S3**. TEM images of the Gd-organic precursors after calcinating at 200 °C (A), 400 °C (B) and 600 °C (C) for 2 h.


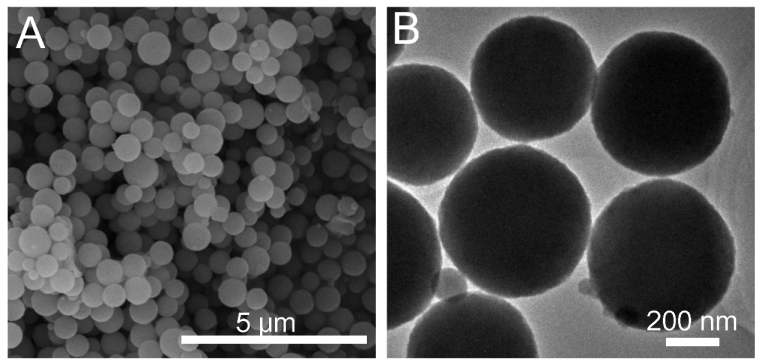


**Figure S4**. (A) SEM and (B) TEM images of spherical 5% Eu-doped Gd-organic precursors.

**
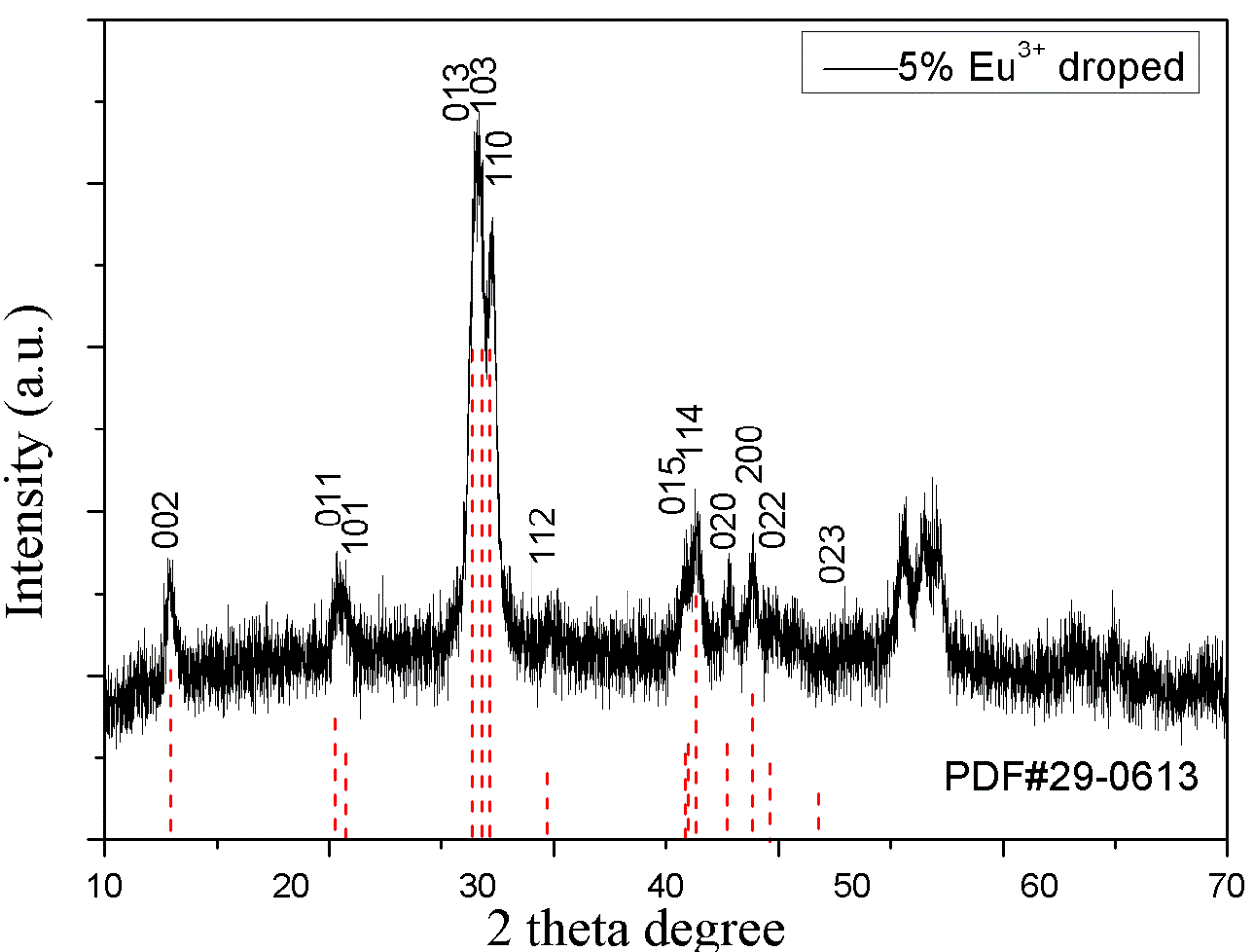
**

**Figure S5**. XRD pattern of 5% Eu-doped Gd_2_O_2_SO_4_ hollow spheres.

**
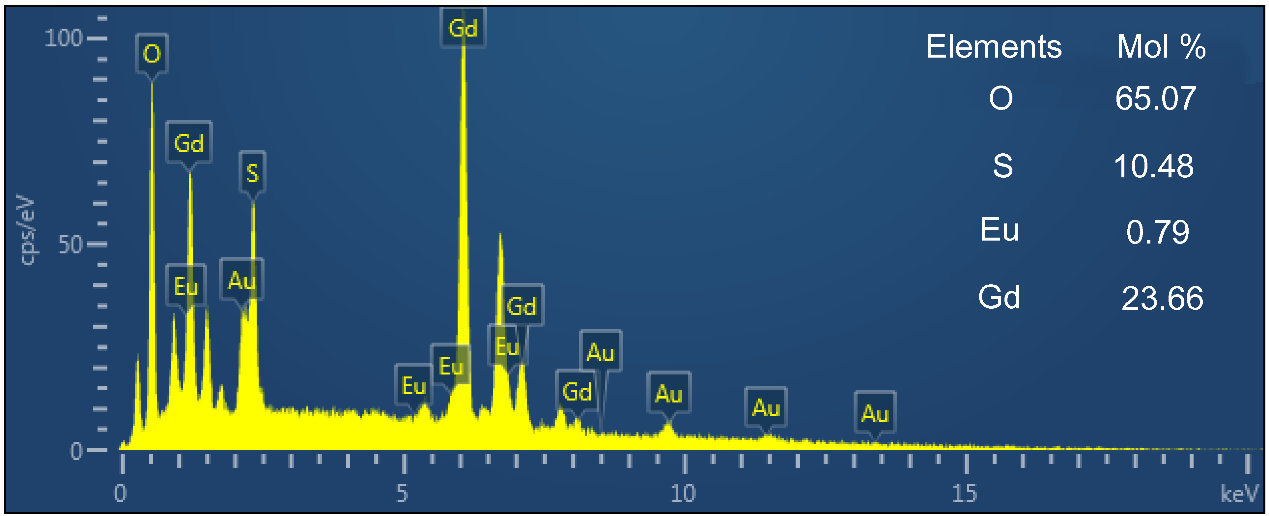
**

**Figure S6**. EDS pattern of as-prepared 5% Eu-doped Gd_2_O_2_SO_4_ hollow spheres.


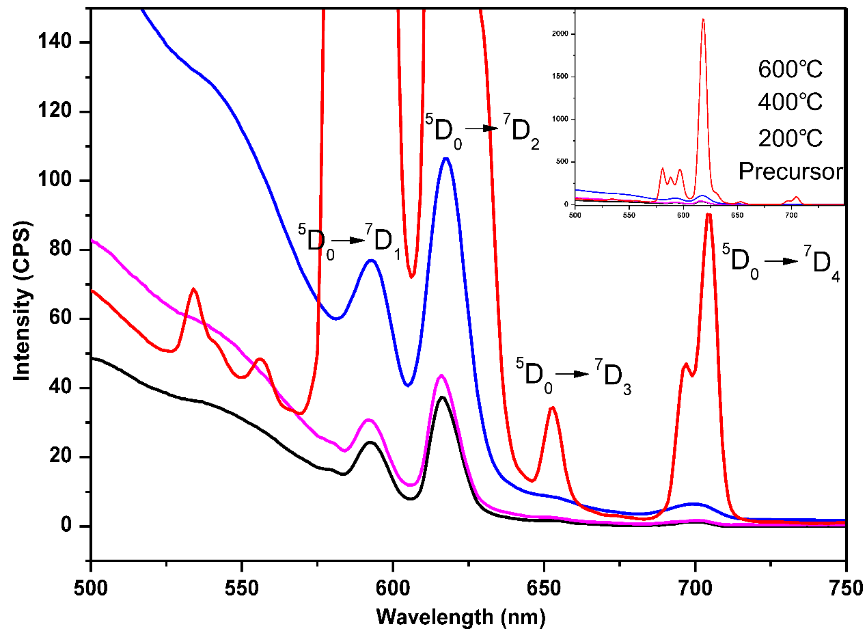


**Figure S7**. Partially magnified emission spectrums of the Gd-organic precursors and the powders after calcinating at 200 °C, 400 °C and 600 °C for 2 h under a laser with wavelength of 270 nm on a Hitachi F-2500 at room temperature. The inset depicts the corresponding full emission spectra.
